# Investigating between-group effects of a physical activity intervention across the physical activity intensity spectrum using multivariate pattern analysis

**DOI:** 10.1186/s12889-025-25722-5

**Published:** 2025-11-24

**Authors:** Eivind Aadland, Olav Martin Kvalheim, Elisabeth Straume Haugland, Kristoffer Buene Vabø, Katrine Nyvoll Aadland

**Affiliations:** 1 Faculty of Education, Arts and Sports, Department of Sport, Food and Natural Sciences, Western Norway University of Applied Sciences, Campus Sogndal, Box 133, Sogndal, 6851 Norway; 2 Department of Chemistry, University of Bergen, Box 7800, Bergen, 5020 Norway

**Keywords:** Children, Preschoolers, Accelerometer, Cluster RCT, Multivariate pattern analysis

## Abstract

**Background:**

Effects of physical activity (PA) interventions are evaluated in many settings, but novel methods are needed to determine effects of interventions across the PA intensity spectrum. The aim of this study was to test the applicability of multivariate pattern analysis to determine between-group effects of an intervention study across the PA intensity spectrum.

**Methods:**

A sample of 698 Norwegian preschool children (mean age 3.8 years) from the Active Learning Norwegian Preschool(er)s cluster randomized controlled trial provided data on PA (ActiGraph GT3X+) before and after short- (7 months) and/or long-term (18 months) follow-ups after a preschool PA intervention. Multivariate pattern analysis and linear mixed models were used to determine effects across the intensity spectrum using PA descriptors of different resolutions (4, 17 and 51 variables).

**Results:**

Higher-resolution PA descriptors led to marginally better model fit than lower-resolution descriptors in multivariate pattern analysis (explained variances 1.27–3.59%). We detected significant standardized mean differences of ± 0.15–0.36, with results for multivariate pattern analysis and linear mixed models being comparable.

**Conclusion:**

We discuss applicability of these approaches for analysis of between-group effects on PA. We conclude that multivariate pattern analysis is a suitable analytic approach to evaluate between-group effect patterns across the PA intensity spectrum of intervention studies, but suggest it being a secondary approach for cluster trials given challenges of appropriately taking clustering into account.

**Trial registration:**

Clinicaltrials.gov, identifier NCT04048967, registered August 7, 2019, (https://clinicaltrials.gov/ct2/show/NCT04048967?term=actnow&rank=1).

**Supplementary Information:**

The online version contains supplementary material available at 10.1186/s12889-025-25722-5.

## Introduction

Given the favourable effects of physical activity (PA) on several health and developmental outcomes and PA action plans and guidelines encouraging increased efforts to increase PA levels [1–3], PA interventions are incorporated and evaluated in many settings. Aims of such interventions could be to reduce sedentary time (SED) and/or increase time spent in one or more PA intensities, such as light intensity PA (LPA), moderate intensity PA (MPA) and/or vigorous intensity PA (VPA), or to increase time spent in higher in favour of lower PA intensities. Because of the correlated nature of these variables constituting the intensity spectrum, several intensity variables will be affected [4], irrespective of the specific study aim. Therefore, it should be favourable to determine this pattern of effects across multiple intensities.

Testing of intervention effects on PA and/or SED are traditionally carried out by testing effects on separate intensity variables, although a compositional data analysis approach has been used to evaluate combined effects across intensities [5, 6]. Multivariate pattern analysis was introduced to the field of PA epidemiology in 2018 to assess associations for the accelerometer-determined PA intensity spectrum with health and development outcomes [4, 7–9]. This analytic approach can model any number of highly correlated explanatory variables using latent variable modelling [10, 11]. This feature allows use of uniaxial or triaxial PA descriptors with higher resolution (typically 16–99 intensity variables [7, 8, 12–14]) than the traditional PA descriptor (typically SED, LPA, MPA, VPA and/or moderate to vigorous intensity PA (MVPA)). This approach has been shown to strengthen associations with outcomes. For example, in schoolchildren [4, 8], the explained variance increased from 10 to 17 to 30% when cross-sectionally regressing a cardiometabolic composite score on a traditional PA descriptor, a high-resolution uniaxial descriptor or a high-resolution triaxial descriptor, respectively. Since intervention effects on PA often are small [15–18], multivariate pattern analysis should be well suited to detect between-group differences by allowing inclusion of richer data and by exploiting the correlated nature of these data. The results by Fridolfsson et al. [19] support this notion, showing significant association patterns for the PA intensity spectrum across group and time when using multivariate pattern analysis, but not when using univariate statistics. However, we are not aware of any studies that have investigated patterns of between-group intervention effects across the PA intensity spectrum using multivariate pattern analysis.

We recently showed favourable, small intervention effects of the cluster randomized controlled Active Learning Norwegian Preschool(er)s (ACTNOW) study on PA, evaluating effects of a multicomponent PA intervention in preschoolers using a traditional univariate analytic approach [20]. Standardized mean differences (SMDs) for PA during preschool hours were −0.18–0.16 (−4.4–2.1 min/day) in intention-to-treat analyses and −0.35–0.33 (−8.4–4.1 min/day) in per-protocol analyses. These effects were derived applying linear mixed model regression to a traditional PA descriptor, while it is unknown how jointly analysing the full intensity spectrum with higher resolution PA descriptors may affect the findings.

The aim of the present study was to test applicability of multivariate pattern analysis to determine patterns of effect of the ACTNOW study across the PA intensity spectrum using multiple PA descriptors and compare findings for this approach with findings from a linear mixed model. We hypothesized that 1) models for multivariate pattern analysis would improve when using richer data (i.e., when using more variables to describe the intensity spectrum and using triaxial versus uniaxial data) and 2) that multivariate pattern analysis would be a stronger approach than linear mixed model to reveal effects since it exploits the richer data and its correlated nature.

## Methods

### Design and sample

The present study used baseline, short- (7 months) and long-term (18 months) follow-up data from the ACTNOW study [21]. ACTNOW was a cluster randomized controlled trial investigating effects of a multicomponent preschool PA intervention in Sogn og Fjordane County, a rural area in western Norway, conducted between September 2019 and June 2022. The study was two-armed (intervention and control; 1:1 ratio), used random allocation at the preschool level and was designed to detect statistically significant SMDs of 0.25–0.30 [21]. A total of 1265 preschool children aged 2.7–6.6 years (born in 2014–2017) from 46 preschools (response rate of preschools: 82%; response rate of children: 83%) participated in the study. Since we included both short- and long-term follow-up in this study, 822 children aged 3–4 years at baseline were eligible for inclusion in the analyses (i.e., 5-year-olds were enrolled in school after 7 months).

Preschools received information and agreed to participate in the study. Parents of all participating children received oral and written information about the study and provided written consent prior to testing. We explained the procedures according to the children’s level of understanding. The institutional ethics committee and the Norwegian Centre for Research Data approved the study (reference number 248220). The study is registered in clinicaltrials.gov August 7, 2019, with identifier NCT04048967 (https://clinicaltrials.gov/ct2/show/NCT04048967?term=actnow&rank=1).

### Intervention

The intervention conceptually had two levels: *the preschool level* (professional development) and *the child level* (physical activity), placing the preschool as an influential institution for child development according to a socioecological model [22]. From each preschool, the director and a minimum of one teacher per department (77 staff members in total) participated in a 7-month professional development structured as a 15-credit education module at master’s level (qualifying for credits were optional).

Details about the intervention have been reported previously [20, 21]. Briefly, at the preschool level, the aim of the professional development was to enhance staff expertise on (1) physically active play and its relevance for child development and (2) planning and implementation of the ACTNOW-intervention within the preschool, to increase capacity to intervene at the child level. The professional development was approximately 50 h in total, consisting of five face-to-face seminars, three webinars, and nine digital lectures. Additionally, a one-day booster session was held one year after startup. At the child level, the intervention consisted of four core components delivered by the preschool staff with the aim of promoting whole-child development: 1) MVPA (60 min/day), 2) motor challenging PA (90 min/week), 3) cognitively engaging PA play (90 min/week), and 4) physically active learning (90 min/week). These components were delivered in different ways, from child-initiated or directed free play to structured staff-led activities.

We used a flexible intervention approach where the intervention was co-created with staff and tailored to their unique contextual factors, as recommended in educational contexts [23]. Intervention preschools received tools consisting of portable play equipment of approximately 500 Euro per department, and an online PA toolbox consisting of various activities and resources (https://activeinpreschool.com/). Control preschools were encouraged to continue their normal practices and received the portable equipment and access to the online toolbox after the study was completed.

### Procedures

#### Physical activity measurement

PA was measured using ActiGraph GT3X + accelerometers (ActiGraph, LLC, Pensacola, Florida, USA) [24]. Children wore the accelerometer in an elastic belt on their right hip and were instructed to always wear the monitor for 7 consecutive days, except during water-based activities. Units were initialized at a sampling rate of 30 Hz and files were analysed restricted to hours 08:30 to 15:30 (i.e., preschool hours) using a custom-made code in MATLAB. We used a 1-second epoch to avoid misclassification of PA intensities, in particular to capture high intensity PA correctly [25]. Periods of ≥ 20 min of zero counts were defined as non-wear time [26]. We applied wear time requirements of ≥ 5 h/day and ≥ 3 weekdays to constitute a valid measurement [27].

We included 3 PA descriptors of different resolution for a comparison of their performance for determination of effects across the PA spectrum: A triaxial intensity spectrum including time (min/day) spent in 17 intensity variables from each axis (from 0 to 99, 100–999, 1000–1999, 2000–2999, …, 14000–14999, to ≥ 15000 cpm) (51 variables), a uniaxial intensity spectrum including the same 17 intensities from the vertical axis only, and a traditional intensity spectrum of time (min/day) spent in 4 intensity variables (SED: ≤ 100 cpm, LPA: 101–2295 cpm, MPA: 2296–4011 cpm, VPA: ≥ 4012 cpm) applied to the vertical axis [28]. Minutes per day spent SED, in LPA, MPA, and VPA [28] were used for descriptive purposes.

#### Anthropometrics and demographics

Body mass was measured to the nearest 0.1 kg using an electronic scale (Seca 899, SECA GmbH, Hamburg, Germany), and height was measured to the nearest 0.1 cm with a portable stadiometer (Seca 217, SECA GmbH). BMI (kg/m^2^) was calculated, and children were classified as normal weight (including underweight), overweight, or obese based on criteria proposed by Cole et al. [29]. Parental socioeconomic status (i.e., the highest education level of mother or father) was assessed using a questionnaire completed by each child’s mother and/or father at baseline (Supplemental file 1).

### Statistical analysis

Children’s characteristics and PA were reported as frequencies, means, and SDs. Multivariate pattern analysis was used to compare effects for the traditional PA descriptor, the high-resolution vertical axis PA descriptor and the high-resolution triaxial PA descriptor to answer hypothesis 1. Effects for the traditional PA descriptor and the high-resolution vertical axis PA descriptor were determined using linear mixed models and multivariate pattern analysis for direct comparison of these statistical approaches to answer hypothesis 2. We conducted both intention-to-treat and per-protocol analyses. Intention-to-treat analyses included all children that provided data at baseline and 7 and/or 18 months, irrespective of whether they received the intervention or not. Per-protocol analyses included intervention preschools that self-reported high or very high integration of the intervention on a 5-point Likert scale from very low to very high integration.

Effects from a linear mixed model were determined for each PA variable separately (outcome) using a repeated measures model: *Y = β*_*0*_ *+ β*_*1*_ *dummytime*_*1*_ *+ β*_*2*_ *dummytime*_*2*_ *+ β*_*3*_ *dummytime*_1_*group + β_*4*_ *dummytime*_2_*group, where time_1_ is 7 months, time_2_ is 18 months, β_3_dummytime_1_*group is the effect at 7 months and β_4_dummytime_2_*group is the effect at 18 months [30]. We adjusted for baseline differences by excluding the main effect of group from the model and adjusted for accelerometer wear time by including it as a main effect. We included random intercepts for child and preschool to take the clustering of observations among individuals and preschools into account. Effect estimates were reported as SMDs, and additionally as minutes per day for the traditional PA descriptor, and 95% confidence intervals (CIs). We also reported intraclass correlation coefficients (ICCs) for the cluster effect of preschools.

Multivariate pattern analysis was applied to the intensity spectra equivalent to its previous applications to accelerometer data [7, 14, 31], except that PA variables were modelled as changes over time (i.e., residuals for 7 and 18 months regressed at baseline). Partial least squares (PLS) regression analyses [10] were used to determine the association pattern between group (outcome) and the change in each of the PA intensity spectra (all 4, 17, or 51 variables included as explanatory variables in one joint model). Each PA descriptor was adjusted for change in wear time between the respective timepoints and baseline total PA levels, as a measure representing a summary of the whole intensity spectrum, using covariate projection [32]. Covariate projection uses PLS regression to model associations between the covariates and all other variables, where the residual variance matrix after this projection is used for further modelling. PLS regression handles completely collinear variables through decomposing the explanatory variables into orthogonal linear combinations (PLS components), while simultaneously maximizing the covariance with the outcome variable [10]. The number of PLS components was cross-validated using Monte Carlo resampling [33] with 1000 repetitions by repeatedly and randomly keeping 50% of the subjects as an external validation set and using a validation threshold of 0.5. For each model, we used target projection [11, 34] followed by reporting of multivariate correlation coefficients to show the importance of each PA intensity variable in the multivariate space [35–37]. SMDs were calculated as multivariate correlation coefficients divided by 0.5 (i.e., the SD of a binary group variable coded 0 and 1) to obtain effect sizes directly interpretable as group differences [38], and additionally as minutes per day for the traditional PA descriptor, by multiplying each PA variable by the SD of the corresponding PA variable. Since adjustment for clustering of observations within preschools is not possible in (the application we used to perform) multivariate pattern analysis, we applied 99% CIs as a conservative approach to compensate for a lack of adjustment of standard errors for clustering.

Linear mixed models were performed using SPSS v. 28 (IBM SPSS Statistics for Windows, Armonk, New York, USA). Multivariate pattern analyses were performed using the packgage *mvpaShiny* v. 0.0.0.9000R [32] in R v. 4.3.2 [39],/RStudio v. 2023.09.1 [40].

## Results

### Children’s characteristics

Of the 822 3- and 4-year-old children that were eligible for the study, 698 (85%) children provided valid data for baseline and 7 and/or 18 months and were included in the analysis. 645 (78%) and 618 (75%) children provided valid data for intention-to-treat analyses of short- (7 months) and long-term (18 months) effects, respectively. 506 (62%) and 488 (59%) children provided valid data for per-protocol analyses of short- and long-term effects, respectively. The children’s characteristics are shown in Table 1. Children’s mean (SD) age was 3.8 (0.6) years and 45.7% was girls. PA levels at baseline and at follow-ups are shown in Table 2. Wear time and wear days were stable over time, while PA generally increased from baseline to 7- and 18-month follow-ups.

**Table 1:** Children’s characteristics. Values are means (SDs) if not otherwise stated

	Intervention	Control
n	320	378
Sex (% girls)	44.7	46.6
Age (years)	3.8 (0.6)	3.8 (0.6)
Body mass (kg)	17.3 (2.5)	17.1 (2.4)
Height (cm)	102.5 (5.5)	102.1 (6.0)
Body mass index (kg/m^2^)	16.4 (1.4)	16.3 (1.4)
Overweight/Obese (%)^1^	15.0/1.6	10.6/2.4
Parental education level (%)		
≤ Upper secondary school	24.4	23.3
University < 4 years	28.4	26.5
University ≥ 4 years	41.6	44.7

*N* = 659–698. ^1^Overweight and obesity status are defined by the Cole et al. [29] criteria

**Table 2:** Children’s physical activity levels. Values are means (SDs)

	Intervention			Control		
	Baseline	7 months	18 months	Baseline	7 months	18 months
n	320	294	286	378	351	332
Wear days (n)	4.9 (0.9)	4.9 (0.9)	4.7 (1.0)	5.3 (0.9)	4.9 (1.0)	5.0 (0.9)
Wear time (min/day)	433 (19)	429 (19)	434 (17)	426 (21)	433 (17)	434 (14)
Overall activity (cpm)	753 (197)	894 (229)	889 (255)	788 (200)	913 (257)	899 (237)
SED (min/day)	284 (23)	268 (27)	280 (29)	278 (24)	273 (25)	277 (22)
LPA (min/day)	101 (15)	106 (16)	98 (16)	98 (14)	105 (16)	101 (14)
MPA (min/day)	24.7 (5.9)	27.2 (6.6)	27.9 (7.0)	24.6 (5.8)	27.3 (6.1)	28.5 (6.0)
VPA (min/day)	22.8 (8.0)	27.5 (9.1)	28.3 (10.0)	24.0 (8.0)	28.1 (9.4)	28.5 (9.3)
MVPA (min/day)	47.5 (13.1)	54.7 (14.3)	56.2 (15.7)	48.6 (12.7)	55.5 (13.7)	57.0 (13.2)

Intensities are defined by the Evenson et al. [28] cut points applied to the vertical axis

*SED* sedentary time, *LPA* light physical activity, *MPA* moderate physical activity, *VPA* vigorous physical activity, *MVPA* moderate-to-vigorous physical activity

### Effects on physical activity

#### Multivariate pattern analysis models

**Fig. 1:**
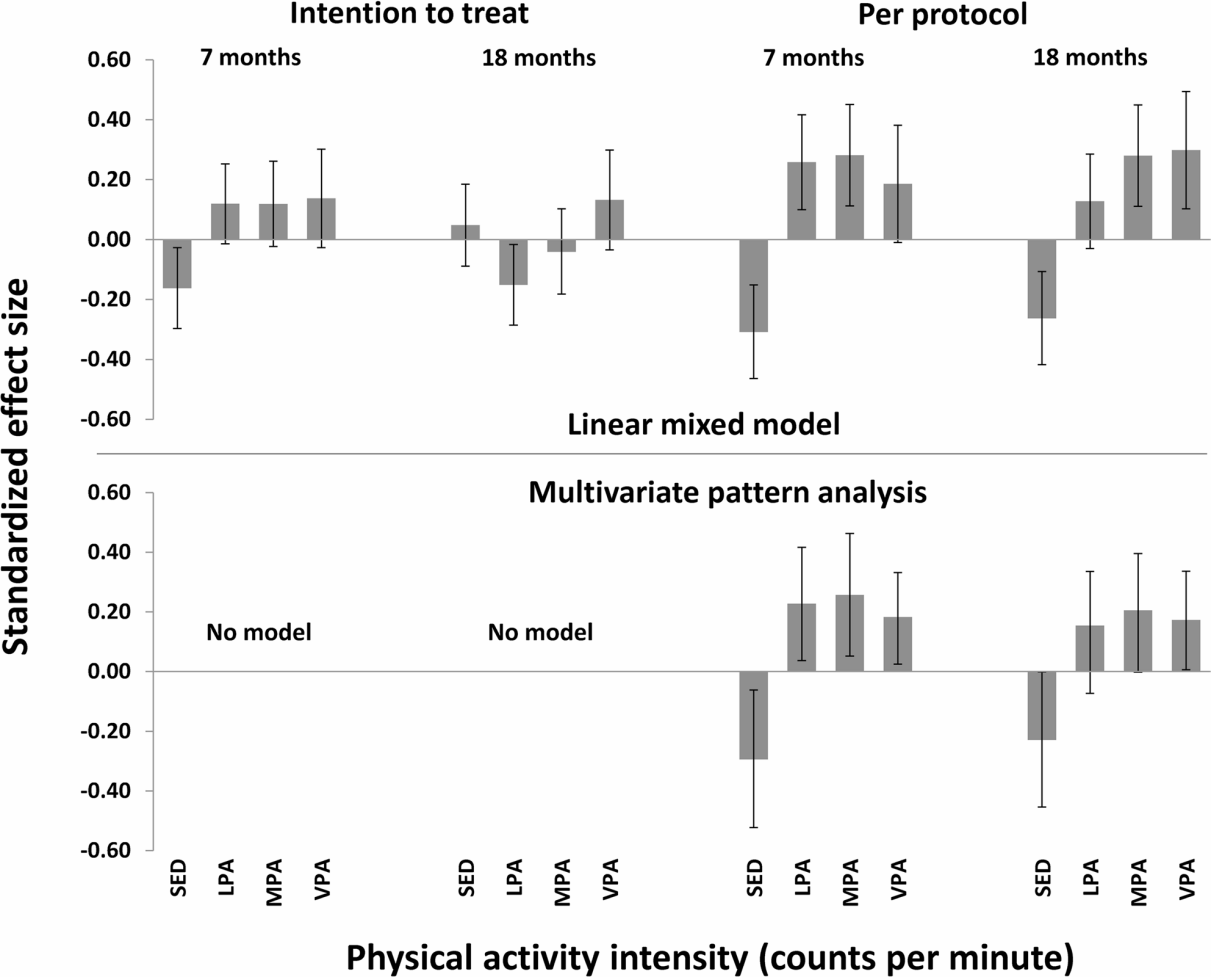
Standardized between-group effect sizes for physical activity using a traditional physical activity descriptor. The upper panel shows effects derived for separate variables using linear mixed models (error bars show 95% confidence intervals with adjustment for clustering among preschools); the lower panel shows effects derived for the variables combined using multivariate pattern analysis (error bars show 99% confidence intervals without adjustment for clustering among preschools)

**Fig. 2:**
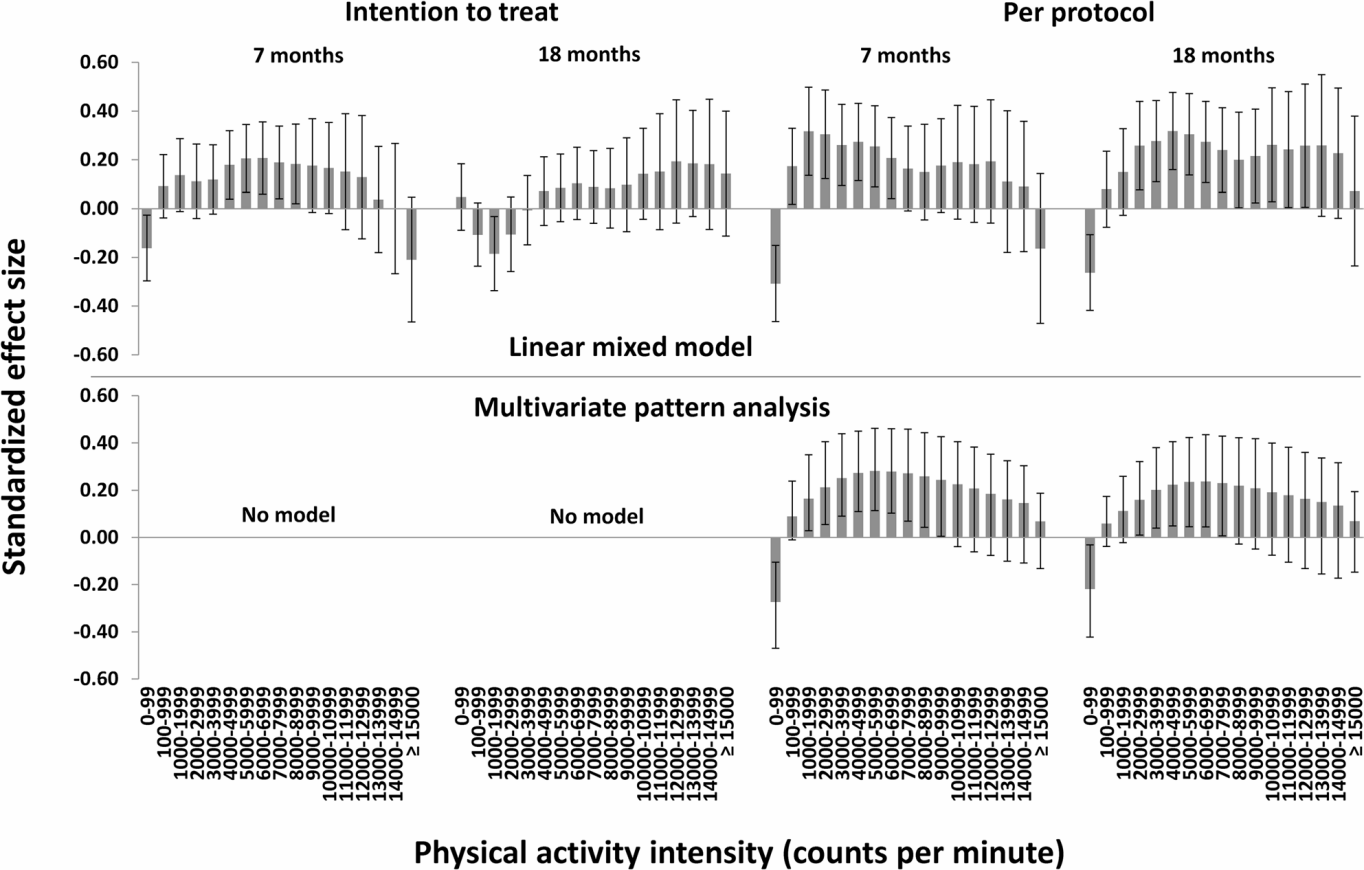
Standardized between-group effect sizes for physical activity using a high-resolution uniaxial physical activity descriptor. The upper panel shows effects derived for separate variables using linear mixed models (error bars show 95% confidence intervals with adjustment for clustering among preschools); the lower panel shows effects derived for the variables combined using multivariate pattern analysis (error bars show 99% confidence intervals without adjustment for clustering among preschools)

**Fig. 3:**
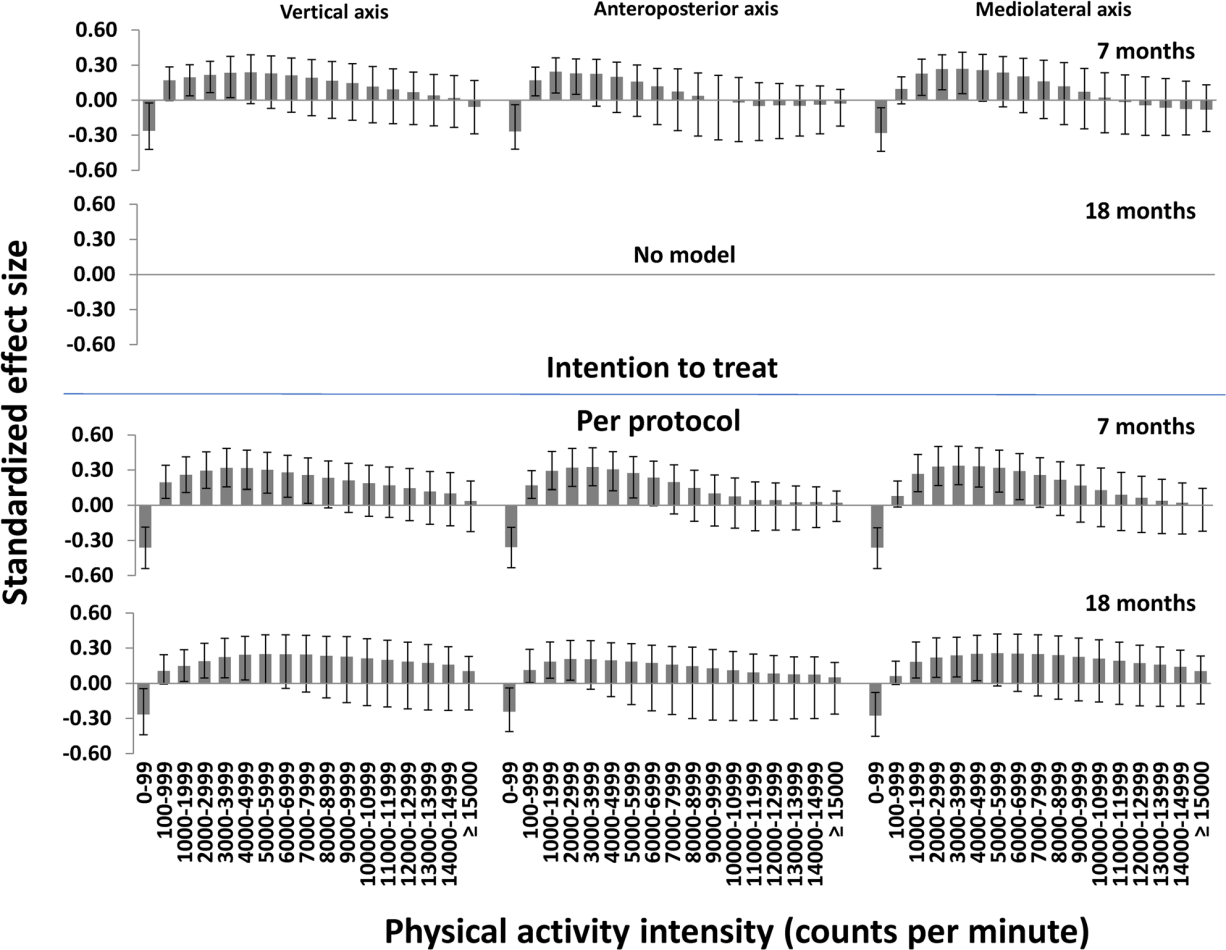
Standardized between-group effect sizes for physical activity using a high-resolution triaxial physical activity descriptor. All panels show effects derived for the variables combined using multivariate pattern analysis (error bars show 99% confidence intervals without adjustment for clustering among preschools)

In intention-to-treat analyses, we found a significant effect for the triaxial PA descriptor over 7 months, but no effects over 7 or 18 months for other PA descriptors (Table 3; Figs. 1, 2 and 3). In per-protocol analyses, we found significant effects for all PA descriptors over 7 and 18 months (Table 3; Figs. 1, 2 and 3). When comparing the significant models for the per-protocol analysis, explained variances were higher for the triaxial intensity spectrum than for the vertical axis intensity spectrum and higher for the vertical axis intensity spectrum than for the traditional intensity spectrum.

**Table 3:** Model fit (explained variances) for multivariate pattern analysis models using various physical activity descriptors

Physical activity descriptor	Intention-to-treat analyses		Per-protocol analyses	
	7 months	18 months	7 months	18 months
Traditional (4 variables)	-	-	2.08	1.27
Specter vertical axis (17 variables)	-	-	2.31	1.42
Specter triaxial (51 variables)	2.37	-	3.59	1.90

-: no predictive PLS regression model

#### Effects on physical activity intensities using different physical activity descriptors

For the traditional PA descriptor (4 intensity variables; Fig. 1; Table 4), we found significant negative effects for SED at 7 months (SMD − 0.16) and LPA at 18 months (SMD − 0.15) while effects for other variables were non-significant in the intention-to-treat analysis using linear mixed models. Models for multivariate pattern analysis were not significant for this descriptor. In the per-protocol analysis, significant negative effects were found for SED in all models (SMDs − 0.31–−0.23), whereas effects for PA were positive with minor differences between intensities in different models (SMDs 0.17–0.30). Effects were rather similar for the two statistical approaches, except for VPA at 7-months (non-significant in the linear mixed model and significant in the multivariate pattern analysis) and MPA at 18-months (significant in the linear mixed model and non-significant in the multivariate pattern analysis). Mean ICC for linear mixed models were 0.08.

**Table 4:** Effects (minutes per day) for the traditional PA descriptor

	Intention-to-treat analyses		Per-protocol analyses	
	7 months	18 months	7 months	18 months
Linear mixed model (mean differences and 95% CIs)				
SED (min/day)	−3.83 (−7.04 to −0.62)	1.13 (−2.09 to 4.36)	−7.29 (−10.99 to −3.58)	−6.21 (−9.90 to 2.51)
LPA (min/day)	1.76 (−0.20 to 3.72)	−2.22 (−4.20 to −0.25)	3.80 (1.47 to 6.13)	1.88 (−0.45 to 4.20)
MPA (min/day)	0.69 (−0.14 to 1.51)	−0.23 (−1.05 to 0.60)	1.63 (0.66 to 2.61)	1.62 (0.65 to 2.59)
VPA (min/day)	1.10 (−0.22 to 2.42)	1.06 (−0.28 to 2.39)	1.49 (−0.09 to 3.07)	2.39 (0.81 to 3.96)
Multivariate pattern analysis (mean differences and 99% CIs)				
SED (min/day)	-	-	−6.96 (−12.39 to −1.47)	−5.42 (−10.75 to −0.04)
LPA (min/day)	-	-	3.35 (0.54 to 6.14)	2.27 (−1.08 to 4.95)
MPA (min/day)	-	-	1.49 (0.30 to 2.68)	1.19 (−0.01 to 2.29)
VPA (min/day)	-	-	1.47 (0.20 to 2.66)	1.39 (0.05 to 2.70)

-: no predictive PLS regression model

For the high-resolution vertical axis PA descriptor (17 intensity variables; Fig. 2), we found a significant negative effect for 0–99 cpm (SMD − 0.16) and significant positive effects for 4000–8999 cpm (SMDs 0.18–0.21) at 7 months and a significant negative effect for 1000–1999 cpm at 18 months (SMD − 0.18), while effects for other variables were non-significant in the intention-to-treat analysis using linear mixed models. Models for multivariate pattern analysis were not significant for this descriptor. In the per-protocol analysis, significant negative effects were found for 0–99 cpm in all models (SMDs − 0.31–−0.23). At 7 months, effects were significantly positive for linear mixed models for intensities 100–6999 cpm (SMDs 0.17–0.32) and for multivariate pattern analysis for 1000–9999 cpm (SMDs 0.16–0.28), while effects for other variables were-non-significant. At 18 months, effects were significantly positive for linear mixed models for intensities 2000–12999 cpm (SMDs 0.20–0.26) and for multivariate pattern analysis for 2000–7999 cpm (SMDs 0.16–0.24), while effects for other variables were-non-significant. Thus, effects were rather similar for the two statistical approaches, where the multivariate pattern analysis revealed significant effects for higher intensities than the linear mixed model at 7-months (up to 9999 vs. 6999 cpm), while the linear mixed model revealed significant effects for higher intensities than multivariate pattern analysis at 18-months (up to 12999 vs. 7999 cpm). Mean ICC for linear mixed models were 0.07.

For the high-resolution triaxial PA descriptor (51 intensity variables; Fig. 3), which were only analysed using multivariate pattern analysis, we found significant negative effects for 0–99 cpm for all axes (SMDs − 0.28–−0.26) and generally significant positive effects for intensities 100–3999 cpm (with minor differences across axes), while effects for other variables were-non-significant, at 7 months (SMDs 0.17–0.24). We found no significant model at 18 months. In the per-protocol analysis, negative effects were found for 0–99 cpm for all axes at both 7 and 18 months (SMDs − 0.36–−0.24). Generally, effects were significantly positive for intensities in the range 100–7999 cpm at 7 months (SMDs 0.19–0.34) and 1000–5999 at 18 months (SMDs 0.15–0.25) (with minor differences across axes), while effects for other variables were non-significant.

## Discussion

In this paper, our aim was to assess the applicability of multivariate pattern analysis to determine patterns of between-group effects of an intervention across the PA intensity spectrum. The main finding was that this method is suitable to determine effect patterns that are easily interpretable. Findings showed that 1) richer data led to improved models (i.e., explained variances) for multivariate pattern analysis, which confirmed our first hypothesis, whereas 2) findings for multivariate pattern analysis and linear mixed models generally were comparable with no obvious improvements in effects from multivariate pattern analysis, which questions our second hypothesis. In the following, we discuss these findings with a focus on the pros and cons of the two analytical approaches.

We were able to detect significant SMDs for PA/SED of ± 0.15–0.36 across intention-to-treat and per-protocol analyses. These findings are consistent with previous studies investigating effects of PA interventions in preschoolers [16, 18]. The mean effect of interventions on MVPA in children aged 0–5 years old across 21 studies meta-analysed by Hnatiuk et al. [16] was 2.88 min per day, which is consistent with the effect shown herein of 3.13 (95% CI 0.92–5.34) minutes per day in intention-to-treat analyses using a linear mixed model (result not shown). Finch et al. [18] found a SMD of 0.28 when meta-analysing 17 preschool studies, but considerably differing effects for studies where interventions were delivered by staff only (SMD 0.27) versus those involving experts (SMD 1.26), and for studies having short (SMD 0.58) versus long (SMD 0.07) intervention periods. Consistent with findings from these staff-led interventions, we found favourable, but mostly non-significant SMDs of 0.00–0.28 (mean 0.12) over 7 months and 0.00–0.19 (mean 0.05) over 18 months across PA descriptors for staff-delivered PA in intention-to-treat analyses. The ACTNOW study was not designed to detect such small effects [21].

Consistent with previous findings for association analyses [4, 8], we hypothesized that multivariate pattern analysis could reveal stronger patterns of effect when including high-resolution (uniaxial or triaxial) PA descriptors compared to a traditional PA descriptor including a few summary intensities. This hypothesis was confirmed. In the intention-to-treat analysis at 7 months, only the triaxial PA descriptor (51 variables) revealed a predictive pattern of effect, while effects for the high-resolution uniaxial PA descriptor (17 variables) and the traditional PA descriptor (4 variables) were non-significant. Additionally, explained variances increased in per-protocol analyses at both timepoints when including higher- versus lower-resolution data. This effect apparently means that fine-grained intensity variables and the anteroposterior and mediolateral axes capture information that is lost when using less comprehensive data. Most PA interventions in preschoolers include many types of active play constituting various intensities and activity patterns (e.g., running, jumping, cycling, climbing, crawling, carrying materials, rough and tumble play). Retaining a detailed intensity distribution and including axes that potentially capture activities that the vertical axis does not, seems sensible and is supported by our data.

Despite having the ability to exploit rich data, multivariate pattern analysis was in some cases less sensitive to detect effects than linear mixed models, thus questioning our second hypothesis. First, we found some significant effects for both the traditional PA descriptor and the high-resolution uniaxial PA descriptor in the intention-to-treat analysis using linear mixed models that was not detected using multivariate pattern analysis because we did not obtain predictive regression models. PLS regression models (i.e., the number of predictive PLS components) are validated using a Monte Carlo resampling procedure repeatedly estimating models in a calibration dataset and evaluating the predictive performance of the models in a validation dataset, where each estimation randomly includes 50% (or any other portion) of the sample to avoid overfitting [33]. The weak models in the intention-to-treat analyses, in this case having explained variances less than 1.27–3.49% (as found for predictive models), was not found significant using this procedure. This is arguably a more conservative approach than using a linear mixed model fitted to the full dataset. Thus, the apparent better sensitivity of the linear mixed model could be a result of overfitting. Second, some variables were significant in linear mixed models, but not in multivariate pattern analysis, particularly in per-protocol analysis at 18 months. Given the cluster design of the ACTNOW study [21], we included random intercepts for preschool in the linear mixed model. ICCs in these analyses (means of 0.07–0.08) was rather similar to the median ICC of 0.05 for preschool-based studies reported in the meta-analysis by Finch et al. [18] and ICCs in a large Norwegian surveillance study (ICCs 0.05–0.12) [41]. Taking clustering into account reduces the effective sample size by increasing standard errors of estimates and is fundamental to arrive at correct ESs in cluster trials [42, 43]. Since we are not aware of suitable approaches to handle clustering in multivariate pattern analysis, we applied 99% CIs to compensate for this effect. Given the relatively low clustering, which only led to minor increases in standard errors in linear mixed models (8.1%, result not reported), using 99% as compared to 95% CIs in multivariate pattern analysis was a conservative choice that increased CIs by 32% (i.e., z-scores 2.58 versus 1.96) and thus increased the risk of type II errors. In trials without clusters, 95% CIs would be an obvious choice, whereas arriving at correct standard errors in cluster trials is challenging. Thus, for cluster trials, we suggest an analytical approach that appropriately take clustering into account should be used as the primary analysis, while multivariate pattern analysis can be applied as a secondary analysis to explore effects across the intensity spectrum. In this case, 95%, 99% or any other CI could be selected a priori based on the prevailing evidence. However, since the aim of this approach would be to explore patterns of effects rather than determine specific effects, choosing the most appropriate correction for clustering may be less important for interpretation. This approach would be consistent with analysing a priori determined specific variables, for example, SED, total PA, or MVPA, depending on study aim, as primary outcomes and other variables as secondary outcomes.

When analysing effects across many variables, a researcher would likely apply the most feasible approach. Multivariate pattern analysis is elegant and less time-consuming as only one model is needed to estimate effects across all variables. This would also avoid potential problems of increased type 1 error rate due to multiple testing in univariate approaches where each variable is tested separately. Correction for multiple testing could be carried out, but such correction is debated [44, 45] and would be conservative, possibly leading to type II errors, when applied in analyses with strongly multicollinear outcome variables. Multivariate pattern analysis protects against chance findings by evaluating the predictive performance of one joint model including all explanatory variables and as discussed above, also include a validation procedure to assess performance in new samples to avoid overfitting.

One conceptual and technical issue when analysing PA effect patterns in multivariate pattern analysis is that regression models must regress group (outcome variable) on change in PA (explanatory variables). This model is contrary to univariate approaches where change in PA (outcome) is regressed on group (explanatory variable). As multivariate pattern analysis can handle binary outcomes, this is not a problem per se, but it means that coefficients need recalculation (i.e., the model needs to be turned around) to be informative. In this case, we derived multivariate correlation coefficients that can be interpreted equivalently to bivariate correlations, though they are derived from the multivariate space [37]. From these, SMDs for each PA variable, equal to Cohen’s d, can be calculated by dividing by SD(x), that is, SD for *group*, which is now treated as the explanatory variable (SD for group is 0.5 since it is a binary variable coded 0 and 1). This approach is similar to standardizing associations for continuous variables given 2 SDs to enable direct comparison of continuous and binary variables in regression analyses [38]. Unstandardized effect sizes can be calculated by subsequently multiplying by SD(y), that is, SDs for *each PA variable*, which is now treated as outcome variables.

### Strengths and limitations

Strengths of this study are the use of a relatively large dataset including 488 to 645 children analyzed across three timepoints including both intention-to-treat and per-protocol analyses. This provided sufficient stability of estimates to identify how findings varied across different analytic approaches and datasets with different effect sizes, and thus confidence in interpretations and conclusions. Findings from multivariate pattern analysis were compared with findings from a linear mixed model. Although effects of interventions can be modelled in multiple ways, linear mixed models including the random effect of preschool is a robust way to estimate effects in cluster randomized designs [30, 42]. Our findings probably generalize to other settings than preschool PA interventions, although intervention and sample characteristics would influence effect patterns. Future studies should investigate how multivariate pattern analysis apply and perform in other settings.

## Conclusion

We conclude that multivariate pattern analysis is a suitable analytic approach to evaluate between-group effect patterns across the PA intensity spectrum of randomized controlled trials. The estimation of effects across all PA variables in one joint model and the use of a Monte Carlo procedure to validate regression models, makes it a conservative approach that protects against chance findings due to multiple testing and overfitting. However, until approaches that can handle clustering are available for multivariate pattern analysis, we suggest multivariate pattern analysis being used as a secondary analytic approach to explore effect patterns across the intensity spectrum.

## Supplementary Information

Supplementary Material 1: Questionnaire used to assess parental education.

## Data Availability

The datasets used and analysed during the current study are available from the corresponding author upon reasonable request.
