# Supplementary material for: Investigating between-group effects of a physical activity intervention across the physical activity intensity spectrum using multivariate pattern analysis
Source: BMC Public Health. 2025 Nov 24;26:3. doi: 10.1186/s12889-025-25722-5 (PMC12763924; doi:10.1186/s12889-025-25722-5)
Supplement: Supplementary file 1 — Supplementary Material 1: Questionnaire used to assess parental education. [file 12889_2025_25722_MOESM1_ESM.pdf]

5. What is your highest completed level of education?

- ☐ Primary school/lower secondary school
- ☐ High school/upper secondary school
- ☐ University, how many years ……………
